# Effects of Time-Restricted Eating on Ketone Metabolism and Immunoregulation in Premenopausal Women: Protocol for a Pilot, Prospective, Single-Arm Dietary Intervention Study

**DOI:** 10.2196/81063

**Published:** 2026-03-06

**Authors:** Geethika P Thota, Natalie A Macheret, Sophia B Glaros, Ila N Kacker, Samson L Cantor, Lilian Mabundo, Noemi Malandrino, Kong Y Chen, Amber B Courville, Sara Turner, Shanna Yang, Andrea Krenek, Thomas C Recupero, Rachael J Klein, Kim Han, Patrycja Puchalska, Peter Crawford, Michael N Sack, Stephanie T Chung

**Affiliations:** 1 Diabetes Endocrinology and Obesity Branch National Institute of Diabetes and Digestive and Kidney Diseases National Institutes of Health Rockville, MD United States; 2 Laboratory of Mitochondrial Biology and Metabolism National Heart Lung and Blood Institute National Institutes of Health Bethesda, MD United States; 3 Clinical and Translational Science Institute University of Minnesota Minneapolis, MN United States

**Keywords:** time-restricted eating, intermittent fasting, ketones, immune cells, immunology

## Abstract

**Background:**

Intermittent fasting interventions, such as time-restricted eating (TRE) without calorie restriction, may offer diverse cardiometabolic health benefits, including reductions in inflammation. However, the underlying metabolic mechanisms are poorly understood.

**Objective:**

This study is designed to evaluate ketone metabolism and immunoregulation during TRE (6-hour feeding and 18-hour fasting) without caloric restriction compared to a conventional dietary regimen (12-hour feeding and 12-hour fasting) in women.

**Methods:**

This is a pilot, prospective, single-arm dietary intervention study in premenopausal women classified as lean and obese, with intraindividual and stratified comparisons. Ketone turnover, immunologic responses, and metabolic profiles will be compared between a conventional dietary regimen (12-hour feeding and 12-hour fasting) at admission and after 3 days of TRE (6-hour feeding and 18-hour fasting).

**Results:**

The trial commenced in August 2024. As of February 2026, participant enrollment is ongoing. Complete enrollment is expected in the December 2026. Integrated analyses will be reported once data analysis is completed, which is expected in the winter of 2027.

**Conclusions:**

This clinical protocol will evaluate whole-body ketone metabolism and immunoregulatory changes in women during TRE using state-of-the-art stable isotope and immunophenotyping techniques. This study will determine the effects of a time-limited dietary pattern, without caloric restriction, on dynamic measures of immunity and metabolism.

**Trial Registration:**

ClinicalTrials.gov NCT06169137; https://clinicaltrials.gov/study/NCT06169137

**International Registered Report Identifier (IRRID):**

DERR1-10.2196/81063

## Introduction

### Background

Time-restricted eating (TRE) is a popular form of intermittent fasting, an eating pattern that alternates between eating and fasting within defined daily eating windows (4-10 h). Early TRE dietary patterns confine eating windows to the daylight waking hours with or without caloric or macronutrient restriction [1]. TRE has been linked to multiple metabolic and immune benefits, including antiaging and longevity benefits [2], healthy body weight, improved insulin sensitivity, and reduced glucose excursions in healthy and overweight individuals [3-11]. TRE has also been associated with changes in thyroid function, menstrual regularity, fatty acid metabolism, and neuroendocrine stress responses, particularly when associated with energy deprivation [12-15].

The underlying mechanisms mediating these metabolic benefits of TRE have not yet been elucidated. Chronic fasting may extend longevity and reduce inflammation [13,14,16]; however, whether shorter fasting durations during TRE modulate immune activation to promote healthy metabolism remains unclear. Fasting-induced ketogenesis during intermittent fasting regimens may be a key mediator of the anti-inflammatory response associated with TRE [17]. Physiological ketosis is protective and homeostatic in contrast to pathological states such as diabetic ketoacidosis [18]. Canonically, ketone bodies are a critical energy source for the brain during periods of diminished glucose availability during prolonged fasting [19]. However, contemporary data suggest that elevated circulating ketone bodies are also key signaling molecules and epigenetic or posttranslational modifiers [19].

Ketone bodies (acetoacetate and β-hydroxybutyrate [βOHB]) interact with various histone deacetylases and other proteins to induce posttranslational modifications, reduce oxidative stress, and inhibit the activation of the nucleotide-binding oligomerization domain–like receptor family pyrin domain containing 3 inflammasome [18,20-22]. Notably, βOHB activates transcription factors and adenosine monophosphate–activated protein kinase signaling, which plays a crucial role in regulating inflammatory pathways [23-25]. During a 24-hour fast in healthy individuals, we demonstrated that fasting blunted inflammatory markers and immune activation in monocytes or macrophages and CD4^+^ T cells both in healthy individuals and in those with mild inflammatory disease (steroid-naive asthma) [26,27]. In a small pilot study of men and women with multiple sclerosis, alternate-day intermittent fasting with caloric restriction was associated with gut microbiome remodeling and immune changes suggestive of reduced interleukin-17 (IL-17)–producing T cells and increased numbers of regulatory T cells [28].

Although prolonged fasting durations have been associated with T cell differentiation, whether the 18-hour fasting component of a TRE dietary pattern would be sufficient to induce changes in ketone metabolism and T cell activation is unexplored. An early 6-hour TRE was associated with increased fat oxidation [5] and increased βOHB concentrations by 0.03 (SE of mean 0.01) mM using a crossover 4-day design [4]; however, no studies have linked ketogenesis during TRE with T cell immunophenotyping. Additionally, variations in the metabolic effects of TRE may be confounded by other biological factors; the ketogenic response to TRE may vary according to biological sex and obesity status. Individuals with obesity, compared to lean individuals, have lower rates of ketogenesis, and this may potentially be influenced by nonalcoholic fatty liver disease [29-31]. Moreover, some studies report higher ketogenesis in women than in men [32-35].

### Research Gap and Significance

No studies have examined the metabolic mechanisms of ketone body metabolism and the anti-inflammatory effects of a 6-hour TRE regimen stratified by sex and obesity status. Given the limited research and discrepant findings regarding the effects of biological sex and obesity on ketone turnover, it is crucial to understand their role in response to dietary interventions. This clinical translational study uses state-of-the-art stable isotope and immune-metabolic phenotyping techniques to determine the impact of shifts in nutrient access and availability on dynamic changes in whole-body and cellular ketone turnover and immune activation in healthy women stratified by obesity status. This protocol complements the previous study conducted in men (NCT04728165) and will be used to explore TRE-mediated immune response in men and women.

### Study Aims

#### Primary Aim 1

The first primary aim is to quantify whole-body βOHB turnover after short-term early 6-hour TRE compared to a conventional 12-hour dietary regimen in women.

#### Primary Aim 2

The second primary aim is to quantify CD4^+^ T cell responses after short-term early 6-hour TRE compared to a conventional 12-hour dietary regimen in women.

#### Secondary Aim 1

The first secondary aim is to quantify changes in postabsorptive glucose rate of appearance (R_a_), measured using (6,6-^2^H_2_) glucose and βOHB R_a_ (intravenous), after 3 days of 6-hour TRE.

#### Secondary Aim 2

The second secondary aim is to quantify CD4^+^ T cell responsiveness (T helper 17 [Th17] polarization) in lean versus obese women after 3 days of 6-hour TRE.

## Methods

### Study Setup

**Figure 1:**
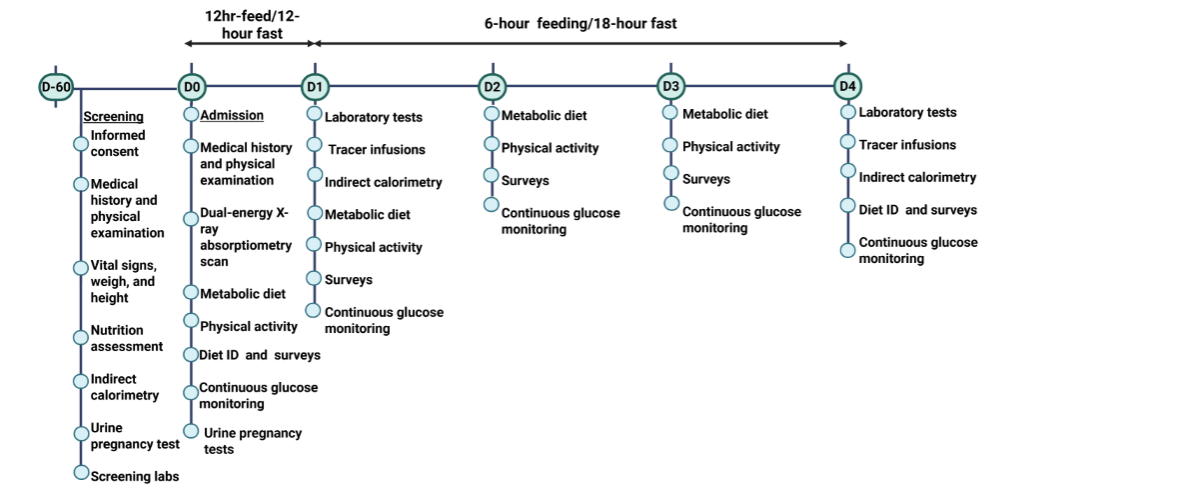
Study schema and timeline. Overview of the study design and timeline across screening, conventional diet, and time-restricted eating phases.

This single-site 5-day inpatient study is a pilot, prospective, single-arm intervention trial in premenopausal women classified as lean or obese, with intraindividual and stratified comparisons. Ketone turnover and immunologic responses will be compared between a conventional dietary regimen (12-hour feeding and 12-hour fasting) at admission and after 3 days of TRE (6-hour feeding and 18-hour fasting; Figure 1). Each participant will serve as their own control, with analyses stratified by obesity classification. We chose a 3-day TRE intervention to determine the acute effects of this dietary pattern on ketone metabolism and immune function. Our previous publications showed that a 3-day TRE in men was feasible and improved glycemic variability and hepatic insulin resistance [36,37]. This study was designed to replicate and validate the 3-day TRE and extend these findings by evaluating glycemia and immunometabolism in women and examining ketone-mediated mechanisms. Secondary measures of glucose metabolism will be compared between groups at the same time points. To explore potential effects on hormonal mediators, circadian rhythms, and cardiometabolic markers, these measures will be evaluated at admission and after intervention. This study will be conducted in the Metabolic Clinical Research Unit at the National Institutes of Health (NIH) Clinical Center, Bethesda, Maryland.

### Participants

**Figure 2:**
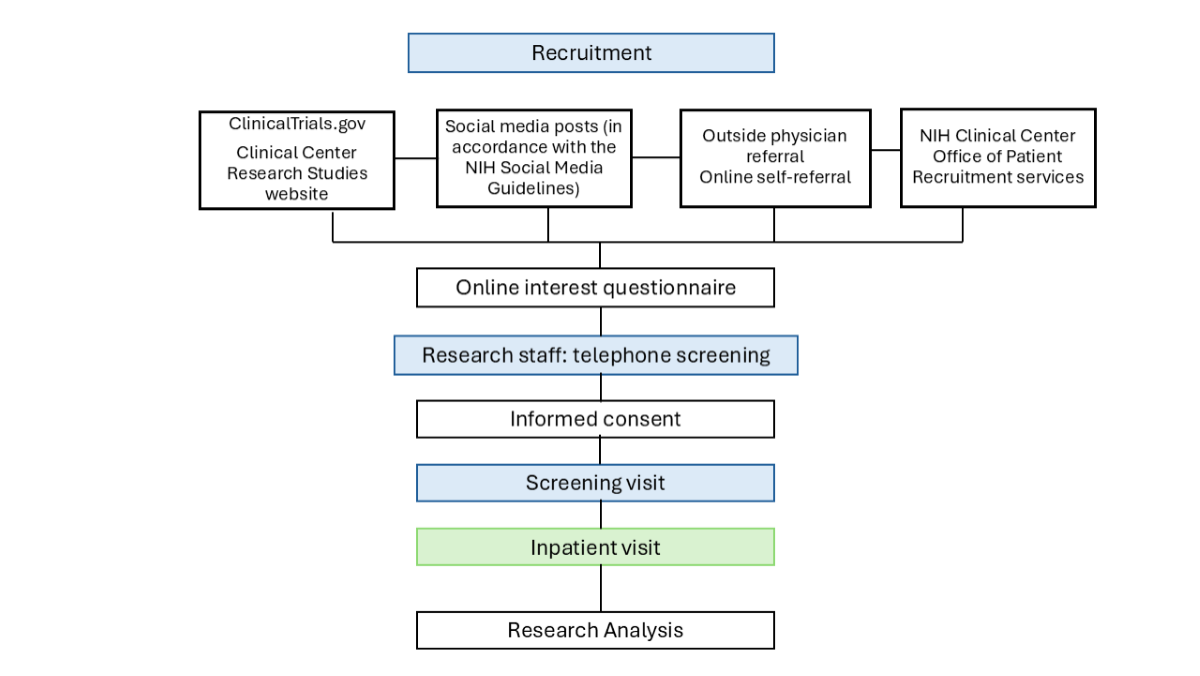
Study flow diagram. Participant recruitment, screening, and study completion. NIH: National Institutes of Health.

Healthy premenopausal women aged 18 to 50 years of all races and ethnicities will be recruited. A full overview of inclusion and exclusion criteria is provided in Textbox 1. An overview of recruitment strategies is provided in Textbox 2. Interested participants will complete an online interest questionnaire and telephone interview before screening. (Figure 2).

**Study inclusion and exclusion criteria.**

**Inclusion criteria**

- Willingness to comply and be available for study procedures
- Women aged between 18 and 50 years in their premenopausal phase (regular menses and follicle-stimulating hormone is below the upper limit of normal)
- BMI of 18 to 24.9 kg/m^2^ or 30 kg/m^2^ or greater
- In good health per medical history and screening laboratory evaluation
- Agreement to adhere to lifestyle considerations throughout the study

**Exclusion criteria**

- Immune, inflammatory, or metabolic conditions that could affect study outcomes, except chronic, controlled hypothyroidism
- Current use of antihyperglycemic medications; systemic steroids; adrenergic stimulants; or drugs affecting sleep, circadian rhythms, or metabolism
- Caffeine intake more than 300 mg daily (approximately three 8-oz cups of coffee)
- Factors affecting circadian rhythms, including shift work, irregular sleep or eating schedules, or fasting for more than 15 hours per day
- History of an eating disorder
- Food allergies, intolerances, or dietary patterns that restrict consumption of the metabolic diet
- Inability to provide informed consent
- Pregnancy or lactation
- Unstable body weight, defined as more than 5% change in the past 3 months
- Competitive sports training or unwilling to follow exercise protocol
- Alcohol intake of more than 3 servings per day
- Current smoker or regular tobacco, vaping, or nicotine use within the past 3 months

**Recruitment strategies.**

**Recruitment strategies**

- ClinicalTrials.gov
- Clinical Center Research Studies website
- Twitter posts and chats with study investigators
- Social media posts in accordance with National Institutes of Health (NIH) Social Media Guidelines
- Use of the NIH Clinical Center Office of Patient Recruitment services, including the creation and distribution of study flyers and dissemination of information through preexisting recruitment avenues, such as the NIH recruitment listserve

### Screening Visit

Study participants will be screened after a 10- to 12-hour overnight fast to confirm eligibility. Screening evaluation will occur within 60 days of the inpatient visit and include obtaining a medical history, physical examination, fasting blood sampling, urine pregnancy testing, anthropometric measurements, resting energy expenditure measured by indirect calorimetry using the ventilated hood technique (Parvo Medics TrueOne 2400), and a nutrition assessment with nutrition research staff. Participants will be asked to complete 3 to 7 days of food logs before their inpatient visit.

The continuous glucose monitoring device (Dexcom, Inc) will either be provided at the screening visit (if eligibility is confirmed) or mailed to the participant approximately 1 week before admission. During the screening visit, participants will receive instructions on how to insert the US Food and Drug Administration–approved continuous glucose monitoring. Device placement instructions will also be reviewed during an in-person or virtual (telehealth) session with a clinician or qualified designee, including guidance on inserting the subcutaneous sensor and attaching the removable transmitter used to measure interstitial glucose levels. Participants will be asked to place the device at home 12 to 24 hours before admission and to wear it throughout their inpatient stay.

### Inpatient Study Visit

Figure 1 outlines the study procedures. On admission to the metabolic unit on day 0, participants will be provided a conventional dietary regimen (09:30 AM to 09:30 PM). A dual-energy X-ray absorptiometry scan (Lunar iDXA; GE HealthCare) will be performed on day 0 to measure fat mass and lean body mass (LBM). Point-of-care ketone levels will be checked every morning using the Precision Xtra Ketone Meter (Abbott Diabetes Care Inc). Surveys will be administered to assess outcomes, including hunger and fullness, appetite, stress, and self-reported positive affect or well-being [38-46]. Participants will have 30 minutes of scheduled light-to-moderate physical activity, involving treadmill walking at a speed calculated to target 35% to 45% of heart rate reserve, using their age-predicted maximum heart rate and resting heart rate obtained during their screening visit.

### TRE Intervention Design

**Figure 3:**
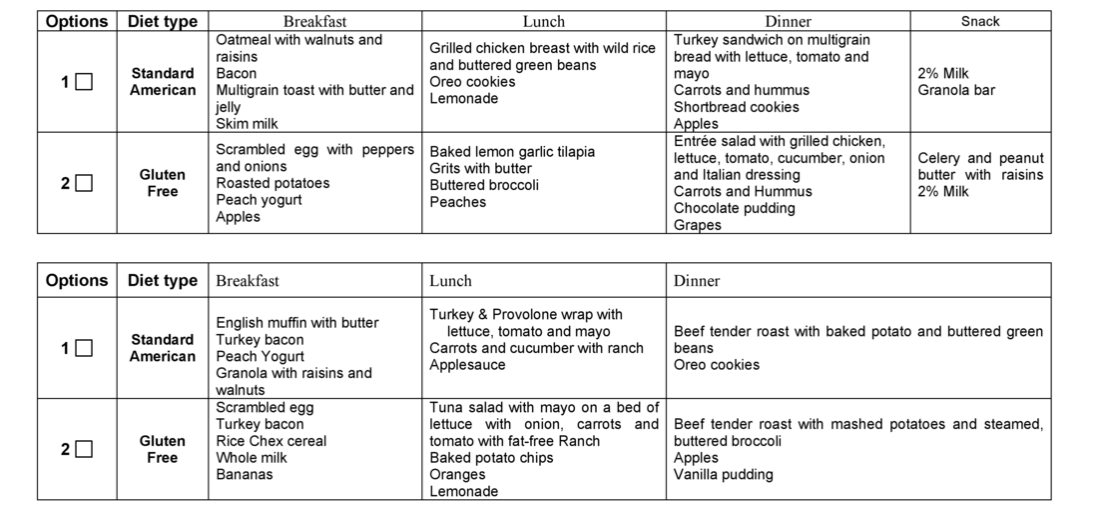
Illustration of a sample menu with available options. (A) Conventional dietary regimen (day 0). (B) Time-restricted eating dietary regimen (days 1-3).

The TRE intervention is a 6-hour early eating window from 09:30 AM to 02:30 PM and will be initiated for 3 consecutive days (days 1-3). Participants will have a 60-minute window to consume each meal (Figure 1). Participants will be asked to consume meals in their entirety, and any foods or beverages not consumed will be reweighed and recorded for data purposes. Eucaloric metabolic breakfast, lunch, and dinner will be provided; each meal is estimated to provide 33% of an individual’s estimated energy needs and a macronutrient distribution of 50% carbohydrate, 35% fat, and 15% protein (Figure 1). Standard breakfast, lunch, and dinner options will be offered from a menu designed by nutrition research team members. Sample menu options are shown in Figure 3.

### Intervention Preparation

Metabolic meals will be prepared at the NIH Clinical Center in the Metabolic Kitchen. Diets will be designed by nutrition research team members using ProNutra diet planning software (Viocare, Inc) to align with a specified macronutrient distribution and total energy provision, informed by indirect calorimetry and an activity factor of 1.4.

### Adherence Monitoring

As an inpatient intervention protocol, this study will monitor and confirm dietary compliance through food weigh backs (as applicable) and detailed case report forms completed by the clinical research study team and clinicians.

### Tracer Administration

**Figure 4:**
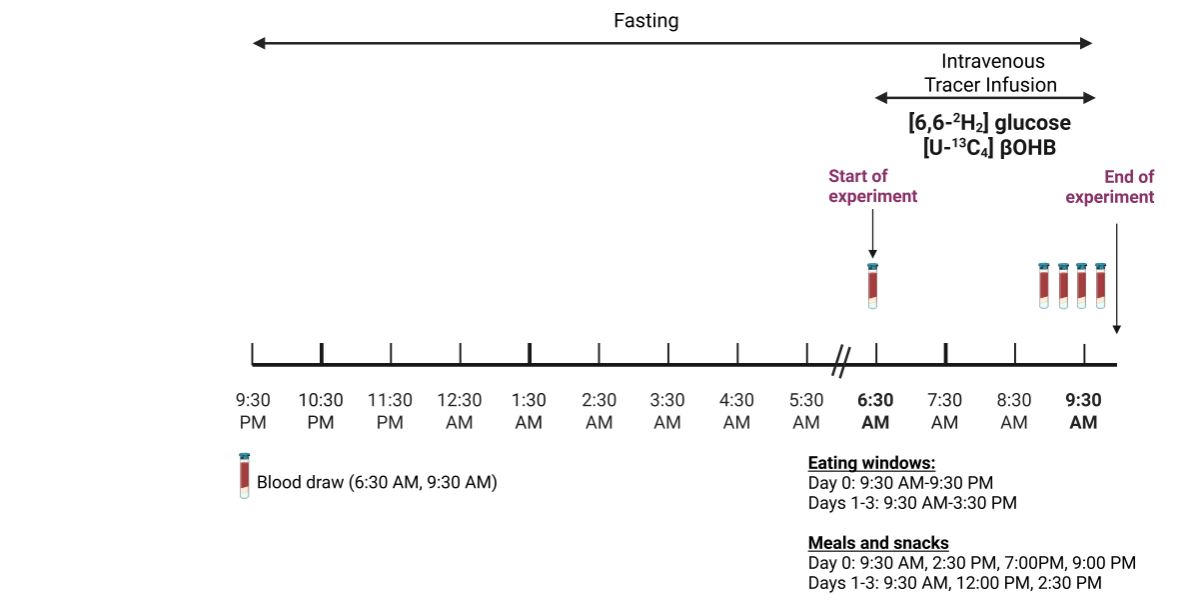
Tracer study protocol. Schematic overview of tracer infusion, blood sampling, and timing relative to the time-restricted eating protocol. βOHB: β-hydroxybutyrate.

On days 1 and 4, participants will receive a fasting 3-hour peripheral infusion of a stable isotope-labeled ketone body and glucose with blood draws obtained at scheduled intervals to trace ketone and glucose metabolism (Figure 4). Ketone body and glucose turnover will be measured using stable isotope tracers. Glucose and βOHB R_a_ will be measured using a primed continuous 3-hour infusion. The (6,6-^2^H_2_) glucose dose will be 0.49 µmol/kg LBM per minute for 180 minutes, with a priming dose of 29.4 µmol/kg LBM, and the (U-^13^C_4_) βOHB will be administered as 1.8 µmol/kg LBM per minute priming dose (10-minute infusion), followed by 0.18 µmol/kg LBM per minute for 170 minutes. Blood samples will be collected before stable isotope administration and during the near–steady-state period (last 30 minutes of the infusion). Whole blood will be centrifuged, and plasma will be transferred and frozen at –80 °C until analysis. Blood samples will be collected for the measurement of glucose, insulin, C-peptide, and ketone bodies. Intravenous stable isotopes will meet NIH minimum standards for stable isotope administration and will be obtained through an NIH-approved pharmacy compounding vendor.

### Sample Size Justification

Sample size determination is based on the ability to detect meaningful differences in ketone-body turnover and CD4^+^ T cell responsiveness and determine effect sizes for short-term TRE. Because calculations are derived from limited past data, variances are anticipated, and the sample size calculations are feasibility estimates.

The primary outcomes are as follows: (1) change in βOHB R_a_ to determine the effect of short-term TRE on ketone-body metabolism (2) change in CD4^+^ T cell responsiveness (T helper type 1, T helper type 2, Th17, and Treg cytokines)

These outcomes will be evaluated using a 2-sided paired Student *t* test (2-tailed) with an α of .05. For primary outcome 1, based on unpublished data (Crawford, M, personal communication, March 2025) in studies of human participants (βOHB whole-body R_a_: 10.4, SD 3.16 µmol/kg LBM/min). A sample of 15 participants has 80% power to detect a difference of 2.5 µmol/kg LBM per minute (25%), assuming a 2-sided α of .05, an SD of 3.16, and a correlation of 0.50.

For primary outcome 2, based on a study of 13 healthy volunteers conducted at the NIH Clinical Center (ClinicalTrials.gov NCT02719899) in which isolated CD4^+^ T cells were investigated, the mean paired refed–fasting differences in IL-17 release were 187.1 (SD 398.79) pg/mL, respectively [21]. Using a 2-tailed paired *t* test with an alpha of .05 (n=15), we will have 80% power, assuming an SD of the difference of 400 pg/mL to detect a clinically important TRE-induced change from admission in IL-17 of 311 pg/mL. We will attempt to recruit and screen in person up to 150 women and enroll 30 women to complete the study, including 15 lean women and 15 women with obesity.

### Data Collection and Statistical Analysis

#### Data Collection Methods

Clinical data (including adverse events, concomitant medications, and expected adverse reactions) and clinical laboratory data will be entered into the Clinical Research Information System, a 21 Code of Federal Regulations Part 11–compliant data capture system provided by the NIH as well as REDCap (Research Electronic Data Capture; Vanderbilt University), a data management system provided by the National Institute of Diabetes and Digestive and Kidney Diseases. These data systems include password protection and controlled access.

#### Outcome Analysis

Data will be analyzed with a statistical software package (eg, Stata [version 18; StataCorp]), with descriptive statistics presented as means (95% CIs), medians (IQRs), or percentages. Due to the pilot nature of this study, no prespecified covariates are assigned. Normality of data will be checked using the Shapiro-Wilk test and visually inspected using histograms. If severe violations of data normality are observed, transformations such as logarithmic or square-root transformations will be applied. If such transformations fail to generate normal data, we will pursue statistical analyses using nonparametric tests, which are conservative but less powerful.

Analysis of glucose levels, glycemic responses, and metabolic rates will be compared using paired 2-tailed Student *t* tests for within-group comparisons or unpaired 2-tailed Student *t* tests for between-group comparisons (lean women versus women with obesity). Statistical significance will be tested at an α level of .05. Quality control procedures will be implemented beginning with the data entry system, and data quality control checks will be run on the database. Any missing data or anomalies will be communicated to the relevant departments for clarification or resolution.

Study procedures will be subject to audits and monitoring visits to ensure compliance with the protocol and applicable regulatory requirements, consistent with the quality assurance program plan. Audit or monitoring visit results will be reported to the principal investigator for further reporting, as appropriate. Study documents and pertinent hospital or clinical records will be reviewed to verify that the conduct of the study is consistent with the protocol plan.

Circumstances that may warrant study termination or suspension include, but are not limited to, the following:

- Identification of unexpected, significant, or unacceptable risks to participants
- Demonstration of efficacy that would warrant stopping
- Insufficient compliance with protocol requirements
- Data that are insufficiently complete and evaluable
- Determination that the primary end point has been met
- Determination of futility

If the study is halted, it may resume once concerns about safety, protocol compliance, and data quality are addressed and approval is obtained from the institutional review board and study sponsor. The study will comply with the NIH Data Management and Sharing Policy, which applies to all new and ongoing NIH-funded research with a clinical protocol that undergoes scientific review.

#### Isotopic Calculations

Rates of glucose and βOHB appearance (R_a_) will be calculated under near–steady-state conditions using the average enrichment of (6, 6-^2^H_2_) glucose or (U-^13^C_4_) βOHB in the systemic circulation using conventional isotope dilution calculations [22].



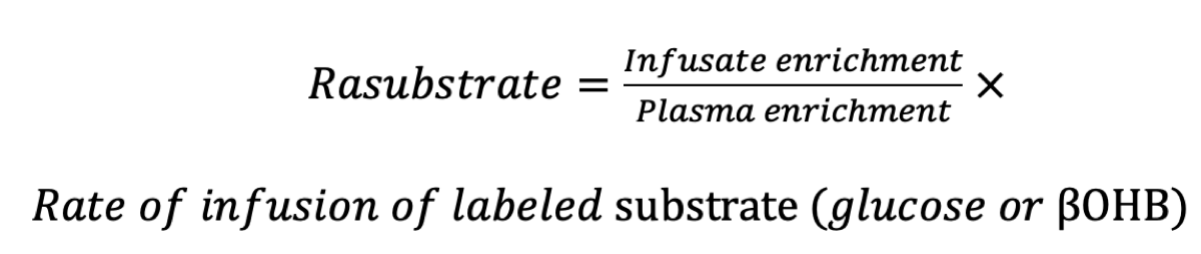



**(1)**

Under fasting and steady-state conditions, R_a_ of glucose reflects hepatic glucose production, and R_a_ of βOHB reflects whole-body βOHB turnover.

Glucose production rate is the sum of the rates of gluconeogenesis and glycogenolysis and is calculated as follows:

Glucose production rate = R_a_ (total) – rate of infusion of labeled glucose

#### Immuno-Metabolomic Profiling and Study Design

The ex vivo metabolic substrate analysis in CD4^+^ T cells will help corroborate the mechanistic insights gained from the clinical study. For cell culture and ex vivo analyses, blood will be drawn immediately before the infusion of isotope tracers on days 1 and 3 after TRE on day 4 (Figure 4). CD4^+^ T cells will be extracted from peripheral blood mononuclear cells (PBMCs) by negative selection using a CD4^+^ T cell Isolation Kit (Miltenyi Biotech) and divided into cells for analysis using mass spectrometry (stable isotope tracing using [U-^13^C_4_] acetoacetate and D-[U-^13^C_4_] βOHB incubations in cell culture and measurement of energy adenylates), immunologic assays (Th17 responsiveness and flow cytometry), and for RNA and protein interrogation of ketolysis and Th17 regulatory pathways.

### Patient and Public Involvement

Patients and members of the public were not involved in the development of the research question, study design, outcome measures, or recruitment plans for this study protocol.

### Ethical Considerations

This pilot study is registered on ClinicalTrials.gov (NCT06169137) and has received approval from the institutional review board of the National Institutes of Health (IRB001746) at the study site. Informed consent will be obtained from eligible and interested participants before enrollment and the start of study procedures. All study data will be deidentified by removal of direct identifiers, including names, addresses, dates of birth, phone numbers, social security numbers, email addresses, medical record numbers, and institutional identification numbers. Each participant will be assigned a unique study identification code based on order of enrollment. Participants will be informed that participation is voluntary and that they may withdraw at any time without impact on their clinical care at the institution. Because there is no direct clinical benefit, participants will receive financial monetary compensation for their time and the inconvenience associated with study procedures.

### Publication Plans

Limited data sharing will be available at the conclusion of the study. Findings from this study will be shared through presentations at national and international scientific conferences and published in peer-reviewed journals.

## Results

Regulatory board approval was obtained in April 2024. Participant screening and enrollment commenced in September 2024 and are ongoing. As of February 2026, 20 participants have been recruited. Data analysis will commence after all participants have been recruited, and results are expected to be published by the winter of 2027.

## Discussion

### Anticipated Findings

To our knowledge, studies have not explored the effects of TRE on the inflammatory and ketone pathways to determine if these factors contribute to improved metabolic health. TRE and intermittent fasting are postulated to induce stress-resistance pathways at integrated metabolic and canonical signaling levels that may be immunomodulatory [2]. Chronic fasting extends longevity and reduces inflammation [13,14,16]; however, whether this occurs with shorter fasting durations during TRE remains unclear. We have previously shown that a prolonged 24-hour fast blunted inflammatory markers and immune activation in monocytes or macrophages and CD4^+^ T cells, both in healthy individuals and in those with a mild inflammatory disease [26,27]. Furthermore, we showed that fasting reduced CD4^+^ T helper type 1 and Th17 polarization and diminished cytokine responsiveness in PBMCs following T cell receptor activation. These findings suggest reprogramming of the PBMCs and differential gene expression changes that were most pronounced in the 24-hour fast versus refed state compared to the 12-hour fast versus refed state [47].

To further determine whether a shorter fasting duration (18 h) will alter T cell activation and metabolism, we conducted a pilot clinical 6-hour TRE trial in men (NCT04728165) at the NIH Clinical Center Metabolic Clinical Research Unit. This study evaluated the effect of a 6-hour TRE versus a conventional 12-hour fasting regimen on immunologic signatures in patients with mild to moderate psoriasis compared to a matched healthy control population. Preliminary data in that trial suggested that 6-hour TRE is sufficient to blunt CD4^+^ T inflammatory responses. This clinical protocol represents the next step in exploring potential mediators of the immunomodulatory response. Key pathways that may influence immunometabolism include fasting-induced ketogenesis, hormonal shifts (fasting-induced low insulin-to-glucagon ratio, increased peptide YY [a satiety hormone], and increased corticosteroid signaling), alterations in microbiome-derived metabolism of short-chain fatty acids, or upregulation of autophagy [48].

### Strengths and Limitations

This translational study integrates clinical and basic scientific research applications. Using a combination of specialized in vivo and ex vivo metabolic deep phenotyping techniques, including stable isotope tracer studies, will elucidate the immunologic signatures of TRE and explore whether obesity is a confounding factor in women. Stable isotope tracer administration will facilitate quantification of ketone production (hepatic) as well as ketone uptake and use by extrahepatic organs and tissues under conditions relevant to the 6-hour TRE intervention. Evaluating the ketogenic response to TRE in women is critical and clinically relevant for understanding the role of biological sex–specific differences in dietary intervention response.

Study limitations include the single-center pilot design, inclusion of women only, and the short study duration, which may limit the generalizability of findings. This is a significant constraint when considering the generalizability of findings to men, older adults, or individuals with metabolic disorders, underscoring the need to address this in a future, larger study with longer-term follow-up. Given the limited sample size and the exploratory nature of the study with intraindividual and stratified comparisons, the results should be interpreted with caution. Findings are intended to be hypothesis generating, which can be further explored in larger and more targeted studies. Future studies should include parallel-arm control design or randomized crossover trials with a conventional 12:12-hour eating schedule.

### Conclusions

This pilot study will be the first to yield generalizable knowledge on whole-body ketone metabolism and immunoregulatory changes in women during early 6-hour TRE. The mechanistic findings will help determine the importance of fasting duration, ketone body production, and immunoregulation to the salutary outcomes of TRE. This analysis will provide much-needed foundational data to inform the design of larger, multicenter randomized trials.

## Abbreviations

βOHB: β-hydroxybutyrate
IL-17: interleukin-17
LBM: lean body mass
NIH: National Institutes of Health
PBMC: peripheral blood mononuclear cell
Ra: rate of appearance
REDCap: Research Electronic Data Capture
TRE: time-restricted eating

## Data Availability

The anonymized dataset and statistical code supporting this study will be made available in a public repository upon study completion and publication.
